# A Dual Diagnostic Dilemma: Viral Encephalitis and Cerebral Venous Thrombosis

**DOI:** 10.7759/cureus.68816

**Published:** 2024-09-06

**Authors:** Adeel Khan, Malik Hasnat ul Hassan Khan, Salman Ullah Khan, Khizar Hayat, Ayesha Khan, Muhammad Arsalan, Muhammad Hamza Mushtaq, Haider Sarfaraz, Muhammad Abbas

**Affiliations:** 1 Internal Medicine, Khyber Medical College, Peshawar, PAK; 2 Internal Medicine, Medical Teaching Institute, Lady Reading Hospital, Peshawar, PAK; 3 Medical Oncology, The Mater Misericordiae University Hospital, Dublin, IRL; 4 Internal Medicine, Naseer Teaching Hospital, Peshawar, PAK

**Keywords:** cerebral venous and dural sinus thrombosis, cerebrovascular injury, herpes simplex virus type 1, meningoencephalitis, viral encephalitis

## Abstract

Herpes encephalitis is caused by herpes simplex virus type 1 (HSV-1) or type 2 (HSV-2). One of the infrequent complications of herpes encephalitis is cerebral venous thrombosis (CVT) because of the inflammation in the brain parenchyma. We report a unique and challenging case of a 14-year-old female patient presenting with confusion, headache, and fever. On examination, there was no neck rigidity and a negative Kernig's sign with no focal neurological deficits. Systemic examination was done to rule out other systems as a cause for her symptoms, and she was empirically treated as a case of encephalitis. An initial computed tomography (CT) scan of the brain without contrast was normal except for a subtle hypoattenuating area involving the right thalamus. Cerebrospinal fluid (CSF) analysis revealed viral infection while we awaited the results of CSF polymerase chain reaction (PCR) and culture analysis for specific microorganisms. Her Glasgow Coma Scale (GCS) deteriorated following an episode of generalized tonic-clonic seizure, and she was subsequently catheterized and an enteral feeding tube (nasogastric tube) was passed. CSF PCR detected HSV-1. Magnetic resonance imaging (MRI) and magnetic resonance venography (MRV) of the brain with contrast revealed encephalitis with superior sagittal sinus, transverse sinus, straight sinus, and vein of Galen thrombosis yielding a diagnosis of HSV encephalitis with concurrent cerebral venous thrombosis. Hence, this required a very specialized and cautious approach to her treatment. She was started on intravenous acyclovir and subcutaneous enoxaparin, and she recovered over the next few days. She did, however, develop acyclovir-induced renal toxicity in the absence of another offending agent, and the dose of the acyclovir was adjusted accordingly. A diagnosis of CVT, although rarely described, should be systematically suspected in patients with HSV encephalitis presenting with sudden deterioration or unexpected neurological findings in the early phase of treatment or inadequate response to treatment for better management and outcomes.

## Introduction

Herpes encephalitis is caused by herpes simplex virus type 1 (HSV-1) or type 2 (HSV-2). HSV-1 and HSV-2 produce a wide variety of illnesses, including central nervous system (CNS) infections such as encephalitis. Herpes simplex encephalitis (HSE) is the most common cause of sporadic, fatal encephalitis in the developed world [1]. HSE is also associated with high morbidity, with only 40-55% of patients capable of resuming activities of daily living after acyclovir treatment. Neurological deterioration or relapse has been reported in 5-26% of cases despite acyclovir treatment [2].

Worldwide, about 60-90% of older adults are seropositive for HSV-1 infection. At the same time, in the younger age group, it is around 54% for HSV-1 [1]. About 30% of HSE is due to primary infection while the remainder is attributed to reactivation or reinfection with HSV-1 [3].

Cerebral venous thrombosis (CVT) is a serious condition characterized by thrombosis in the cerebral venous sinuses and cerebral veins, presenting with symptoms such as headache, altered mental status, seizures, and focal neurological deficits, necessitating immediate medical intervention [4]. The concurrent occurrence of HSV encephalitis and CVT is extremely rare with limited cases described in the literature. This dual pathology poses a challenging case, as both conditions can mimic each other. CVT should be suspected in patients with HSV encephalitis who present with new or unexpected neurologic symptoms (focal neurologic deficits, seizures, altered sensorium) during the early stages of appropriate treatment [2].

This case report discusses a rare HSV encephalitis concurrent with CVT, highlighting the importance of early recognition and comprehensive treatment.

## Case presentation

A 14-year-old female presented to the emergency department with complaints of fever, headache, and odd behavior. She had been running a fever for the last two days; the fever was recorded to be 103° Fahrenheit, continuous, not associated with rigors or chills, and relieved with paracetamol tablets. The headache was continuous, varying between throbbing and dull types involving both sides of the head, with no reducing or exacerbating factors. Her parents reported changes in behavior particularly uttering meaningless words and erratic behavior 12 hours after the onset of fever and headache. Her past medical and surgical history were insignificant.

She was confused on examination and not oriented to time, place, and person. She was febrile and uttering insensible words. On neurological examination, there was no neck stiffness, bilateral plantars were downgoing, Kernig’s sign was negative, and pupils were reactive to light bilaterally. The chest examination revealed normal vesicular breathing with no added breath sounds. The cardiovascular examination had normal s1 and s2 with no added heart sounds. The abdomen was soft and non-tender.

**Figure 1:**
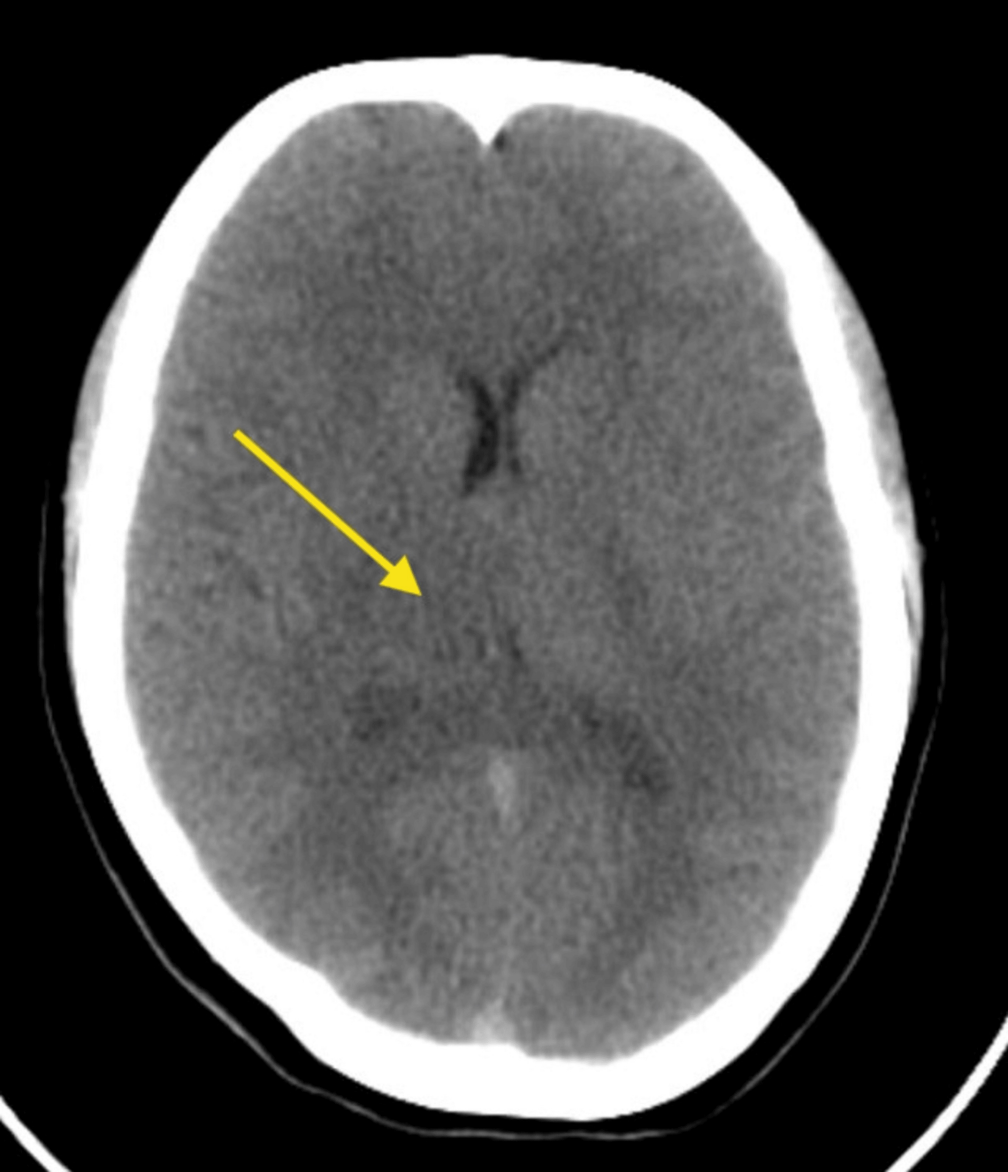
CT brain without contrast A hypoattenuating area involving the right thalamus (yellow arrow)

She was empirically treated as a case of encephalitis. She was started on empiric antibiotics, i.e. injection of ceftriaxone 2 gm IV stat, injection of acyclovir 750 mg IV stat, and injection of artesunate 120 mg IV stat for complicated cerebral malaria, as this area has malaria endemic. Computed tomography (CT) of the brain without contrast was done in the emergency department to rule out any space-occupying lesions or intracranial bleeding, and CT brain demonstrated a subtle hypoattenuating area involving the right thalamus, as shown in Figure 1, raising suspicion for meningoencephalitis.

She developed a single episode of generalized tonic-clonic seizure, which was managed with an injection of diazepam stat and an injection of levetiracetam 500 mg twice daily. Baseline investigations were ordered. A lumbar puncture for CSF analysis was done, and CSF was sent for routine examination (R/E), bacterial culture, acid-fast bacilli, and polymerase chain reaction (PCR) for herpes virus, along with malarial parasite. Cerebrospinal fluid (CSF) analysis indicated viral encephalitis as suggested by the moderately elevated levels of protein, normal glucose level, and presence of lymphocytic pleocytosis as seen in Table 1. It was later confirmed by the detection of HSV-1 in CSF PCR.

**Table 1:** Cerebrospinal fluid analysis, PCR, and culture report

Appearance	Colorless, clear
Protein	112 mg/dl
Glucose	83 mg/dl
RBCs	30
WBCs	8
Lymphocytes	100 %
Neutrophils	0 %
Gram staining	No micro-organism
Viral PCR	HSV-1 Detected
CSF culture	No growth observed

A complete blood picture (CBC) showed neutrophilic leukocytosis and anemia with a hemoglobin level of 7.68 and an MCV of 52. A blood smear was ordered, which reported neutrophilic leukocytosis and microcytic hypochromic anemia. She was started on iron supplements and given a mebendazole dose stat empirically for worm infestation. Her hemoglobin improved as evidenced by serial CBCs shown in Table 2.

**Table 2:** Complete blood picture timeline DOA: day of admission

	1^st^ DOA	4^th^ DOA	6^th^ DOA	8^th^ DOA
WBC (4-11)	11.5	13.8	7.45	7.56
Hemoglobin (11.5-17.5)	7.4 g/dl	7.72 g/dl	9.97 g/dl	10 g/dl
MCV (76-96)	51.7 fl	52 fl	57 fl	60 fl
Platelets (150-450)	310	221	250	247
Neutrophils (40-75%)	78%	82%	80%	65%
Lymphocytes (20-45%)	15%	7%	10%	15%

**Figure 2:**
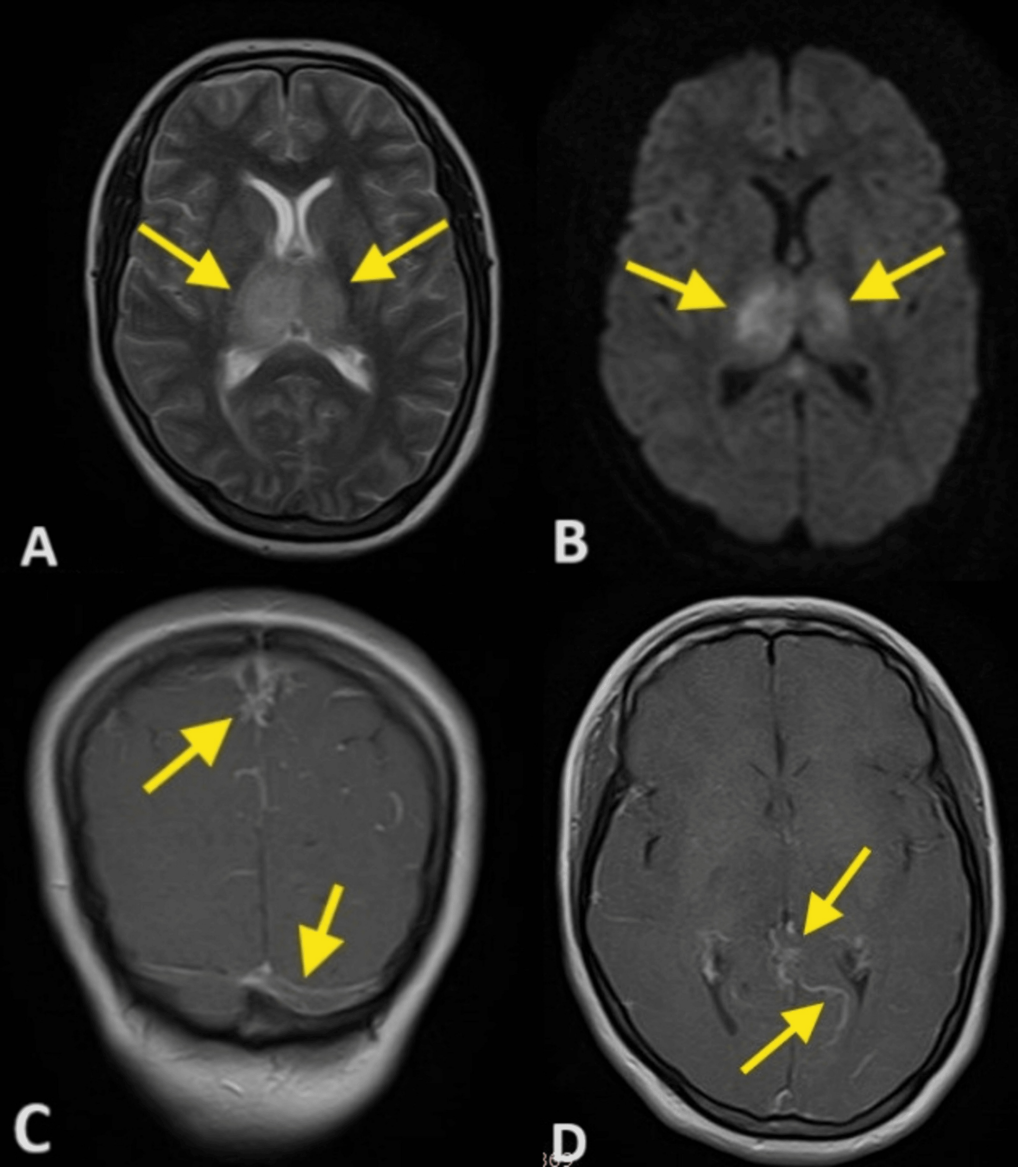
MRI and MRV brain with contrast A: T2-weighted image showing bilateral thalamic hyperintense signals more on the right side (yellow arrows); B: Diffusion-weighted image demonstrating diffusion restriction in bilateral thalamic regions suggestive of thalamic infarcts (yellow arrows); C&D: Post-contrast images showing leptomeningeal enhancement and filling defects in the sagittal and transverse sinus (yellow arrows) MRI: magnetic resonance imaging; MRV: magnetic resonance venography

**Figure 3:**
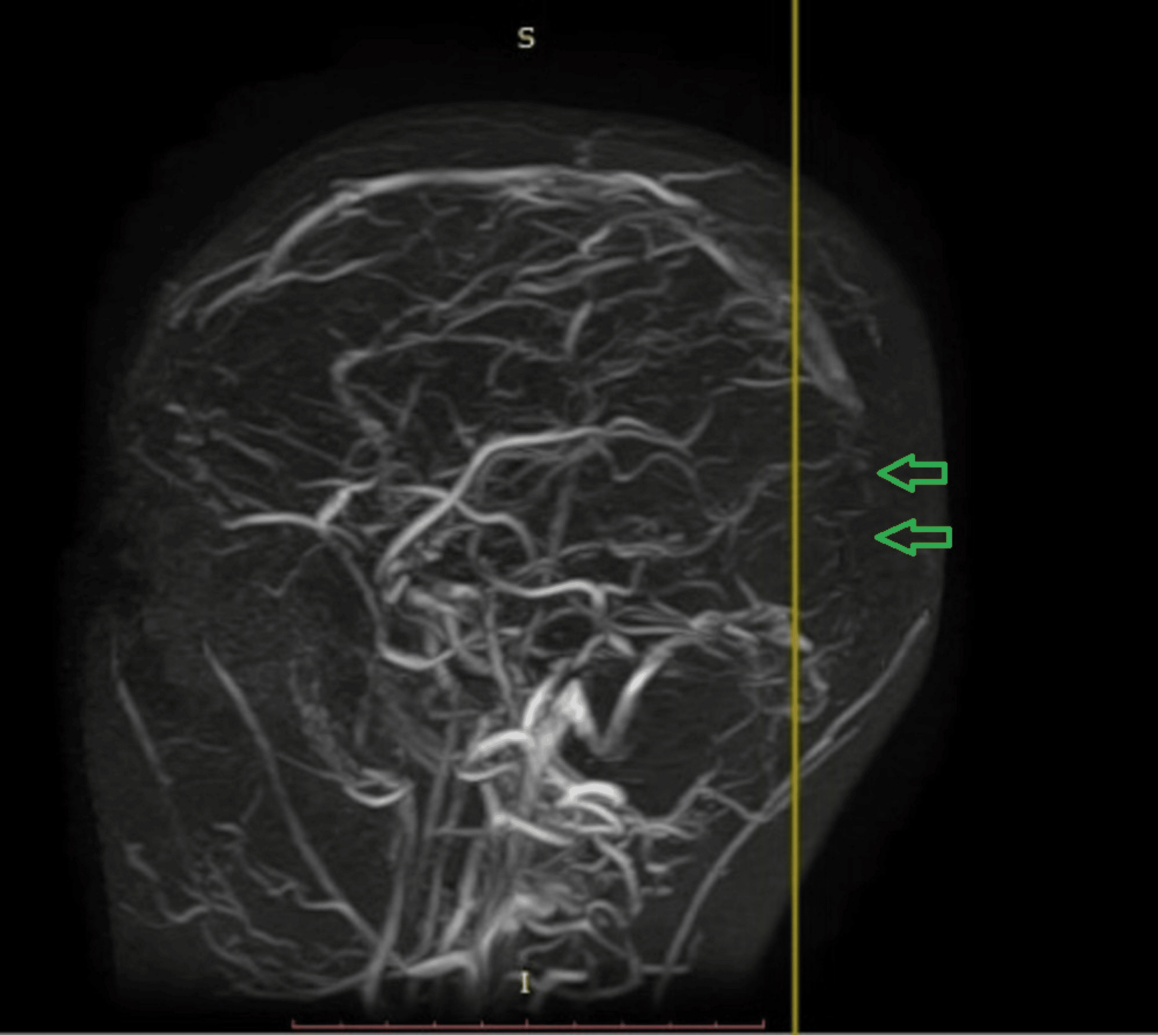
Magnetic resonance venogram of the brain Loss of flow void in the region of the superior sagittal sinus (green arrows), transverse sinus, straight sinus, and vein of Galen suggestive of cerebral venous thrombosis

She was admitted and a magnetic resonance imaging (MRI) of the brain with contrast and a magnetic resonance venogram (MRV) were planned. Brain MRI revealed altered signal intensity changes in the bilateral periventricular areas and thalami on T2-weighted image (T2WI) as seen in Figure 2A, diffusion restriction on the diffusion-weighted image (DWI) suggestive of bilateral thalamic infarct most likely due to involvement of vein of Galen as seen in Figure 2B, and acute lacunar infarcts were noted in higher up sections. Post-contrast images as shown in Figures 2C-2D revealed leptomeningeal enhancement and filling defects in the sagittal and transverse sinus. MRV image shows a filling defect in the superior sagittal sinus, transverse sinus, straight sinus, and vein of Galen, suggesting cerebral venous thrombosis as shown in Figure 3. MRI and MRV brain reported viral encephalitis with concomitant multiple sinus thrombosis, including superior sagittal sinus, transverse sinus, straight sinus, and vein of Galen thrombosis.

On the second day of admission, she was drowsy and confused, with a GCS 11/15, and unable to take oral food. A nasogastric tube was passed to help with nutrition and urinary catheterization was done. MRI brain and CSF analysis reports were available by the third day of her admission, and her treatment was tailored to the radiology and laboratory findings, including Inj. acyclovir 750 mg three times daily + Inj. dexamethasone 8 mg once daily + infusion paracetamol 1 gm three times daily + Inj. enoxaparin 60 mg subcutaneously twice daily + infusion normal saline 500 once daily to help her hydration.

Her clinical condition gradually improved, and by the eighth day of admission, she was oriented in place and person. The nasogastric tube was removed, and she was able to take food orally. She was mobilized, the urinary catheter was removed the next day, and she slowly recovered to her baseline health.

Baseline investigations were repeated daily to monitor the effect of acyclovir on renal function tests. Over the next few days, there was an increase in serum creatinine gradually as shown in Table 3. On the fifth day of admission, serum creatine was 2.79; as a result, a decision was made to decrease the dose of Inj. acyclovir to 500 mg two times daily and infusion of normal saline increased to two times daily. Serum creatinine settled over the next few days and returned to baseline.

**Table 3:** Timeline of renal function tests DOA: day of admission

	1^st^ DOA	5^th^ DOA	6^th^ DOA	7^th^ DOA	9^th^ DOA
Serum Urea (18 -45)	19	77.8	67.56	38	22
Serum Creatinine (0.3-0.9)	0.75	2.79	2.44	1.5	0.61

She was doing much better on the thirteenth day of admission and was discharged home on tablet rivaroxaban 15 mg twice daily for 15 days followed by tablet rivaroxaban 20 mg once daily for 6 months. She was booked for a follow-up in four weeks. On the follow-up appointment, she was healthy, had no complaints, and had no neurological deficits on comprehensive clinical examination.

## Discussion

The central nervous system symptoms of herpes include fever, headache, seizure, focal neurological signs, and impaired consciousness [5]. HSE is a rare manifestation and is associated with significant morbidity, and if left untreated, it can result in a mortality rate of up to 70%[6].

HSV is an unrecognized cause of cerebral venous thrombosis (CVT) [7]. Some studies have suggested that HSE-associated CVT may occur because HSV infection promotes a thrombotic environment [8]. The proposed mechanism for the virus prothrombotic effect is that it possesses procoagulant surface proteins capable of activating the coagulation pathway leading to thrombosis formation [9]. Some studies have also suggested alterations in endothelial cell surface properties [10]. Iatrogenic intracranial hypotension due to lumber puncture has also been a suggested consequence of CVT. In a case report by Vedani et al., it is stated that the lumbar puncture causes a loss in CSF volume and pressure, which are compensated for by an increase in intracranial venous volume, a reduction in flow velocity, and fluid absorption to the cerebral veins, in turn leading to venous stasis.

It can be very challenging to diagnose CVT complicating an HSE. Presenting features of CVT include headaches, which may be unilateral or bilateral. Focal deficit or seizures can occur depending on the type of venous sinus involved [11]. A high degree of clinical suspicion is generally required to make a diagnosis of CVT; typically, patients who rapidly deteriorate on adequate antiviral therapy should prompt a suspicion of underlying venous thrombosis and a quick radiological investigation like MR or CT venography needs to be performed for confirmation.

Once the diagnosis is confirmed, management focuses on addressing life-threatening issues like thrombosis, raised intracranial pressure, seizures, and coma [10]. The mainstay of CVT management is anticoagulation intravenous or low molecular weight heparin followed by oral anticoagulant for 3-6 months in provoked CVT and 6-12 months in unprovoked CVT [12]. Endovascular thrombolysis or thrombectomy in specialist centers may be considered in those cases where anticoagulation is contraindicated or the patient’s symptoms are not improving. [11]. Screening patients for thrombophilias and underlying malignancy is also advised. If the condition is identified and treated early, it can lead to a favorable prognosis.

## Conclusions

Herpes simplex encephalitis (HSE) is a rare but severe condition with a significant risk of cerebral venous thrombosis (CVT), complicating its clinical course. HSV infections may contribute to increased thrombotic risk by several mechanisms, including infecting endothelial cells (EC), leading to a reduction in heparan sulfate proteoglycan synthesis and expression, which in turn prevents antithrombin III from binding to the cell surface. Additionally, the expression of thrombomodulin is diminished, impairing the activation of protein C. Diagnosing CVT in the context of HSE is challenging, often requiring a high index of suspicion and prompt imaging, especially in patients who worsen despite adequate antiviral treatment. Management with anticoagulation is critical, and early intervention can prevent severe complications such as raised intracranial pressure, seizures, and coma. A multidisciplinary approach, including careful monitoring and consideration of advanced therapies, is essential for improving patient outcomes. The case also highlights the importance of monitoring for adverse drug reactions, particularly renal impairment in patients receiving acyclovir.
